# Effects of Spatial Frequency Similarity and Dissimilarity on Contour Integration

**DOI:** 10.1371/journal.pone.0126449

**Published:** 2015-06-09

**Authors:** Malte Persike, Günter Meinhardt

**Affiliations:** Johannes Gutenberg University, Mainz, Germany; Kyoto University, JAPAN

## Abstract

We examined the effects of spatial frequency similarity and dissimilarity on human contour integration under various conditions of uncertainty. Participants performed a temporal 2AFC contour detection task. Spatial frequency jitter up to 3.0 octaves was applied either to background elements, or to contour and background elements, or to none of both. Results converge on four major findings. (1) Contours defined by spatial frequency similarity alone are only scarcely visible, suggesting the absence of specialized cortical routines for shape detection based on spatial frequency similarity. (2) When orientation collinearity and spatial frequency similarity are combined along a contour, performance amplifies far beyond probability summation when compared to the fully heterogenous condition but only to a margin compatible with probability summation when compared to the fully homogenous case. (3) Psychometric functions are steeper but not shifted for homogenous contours in heterogenous backgrounds indicating an advantageous signal-to-noise ratio. The additional similarity cue therefore not so much improves contour detection performance but primarily reduces observer uncertainty about whether a potential candidate is a contour or just a false positive. (4) Contour integration is a broadband mechanism which is only moderately impaired by spatial frequency dissimilarity.

## Introduction

Detection of shapes and objects in complex images requires to group similar elements across space and combine them into larger units. Early attempts to study principles of similarity grouping showed that context is a major determinant for grouping phenomena in various complex stimulus situations [1, 2]. Although this appeared to call for global scale mechanisms, target formation was eventually explained with purely local mechanisms, employing pairs of oriented filters, a rectifier, and a second isotropic filter [3–5]. This simple local mechanism, while capable to explain texture segregation by responding to local differences in orientation and spatial scale, fails to highlight certain types of visual contours in complex images. Such contours are formed by neighboring elements with collinear orientation and become salient to human observers despite strong orientation jitter across the stimulus field. In order to integrate smooth contours in cluttered surrounds a different local principle was required.

The association field model [6] combines the Gestalt rules of similarity and good continuation [7] into a single model to account for fast and effortless detection of non-continuous visual contours in complex surrounds. The association field operates on collinear orientations of disjoint local stimulus elements and allows to integrate these into a contiguous contour percept. Research suggests that the human visual system has developed specialized patterns of neural connectivity to achieve contour integration [8–10]. Interconnections between local orientation detectors are assumed to form the neural basis for contour integration [6, 11, 12]. Lateral connections in the form of inter-columnar synaptic fibres have been found, among others, in the macaque [13, 14], the cat [15], and the tree shrew [16]. Even in early layers of visual cortex, those lateral connections span preferentially between neurons with similar preferred orientations [17], as has been demonstrated for the macaque [18], the cat [15, 19, 20], the owl monkey [21], and the tree shrew [22].

While initial models focused on primary visual cortex (V1) as the locus of contour integration [6, 23–25], many studies since have pointed toward the involvement of higher visual areas. Collinear stimuli induce inter-area coherence of neuronal responses between V1 and V2 [26], and the activation patterns during contour integration are comparable between V1 and V2 [27]. The temporal dynamics of horizontal axonal connections in V1 are moreover similar to those of feedback projections from V2 [28], thus making it difficult to distinguish between V1 or V2 as the likelier substrate for contour integration [29]. Going beyond V2, recent evidence suggests that the lateral occipital complex (LOC) plays a pivotal role in contour integration [30]. Rather than being the source of contour integration, contour related effects found in early visual areas like V1 have even been suspected to be a mere epiphenomenon caused by feedback connections from V4 [31] or LOC [27, 32], where the pooling of information from earlier visual areas constitutes the initial step of contour formation, followed by feedback-induced activation in V1.

Most research about the functional architecture of the association field capitalized on the effect of dissimilarities among contour elements in other features than orientation, thus establishing important findings about functional constraints of the association field. Contour integration is known to be susceptible to flanker orientation [33], element distance [34] as well as size [35], and temporal modulation [36], while it has proven robust against disparities in hue [37], depth [38], and spatial scale [39].

**Fig 1:**
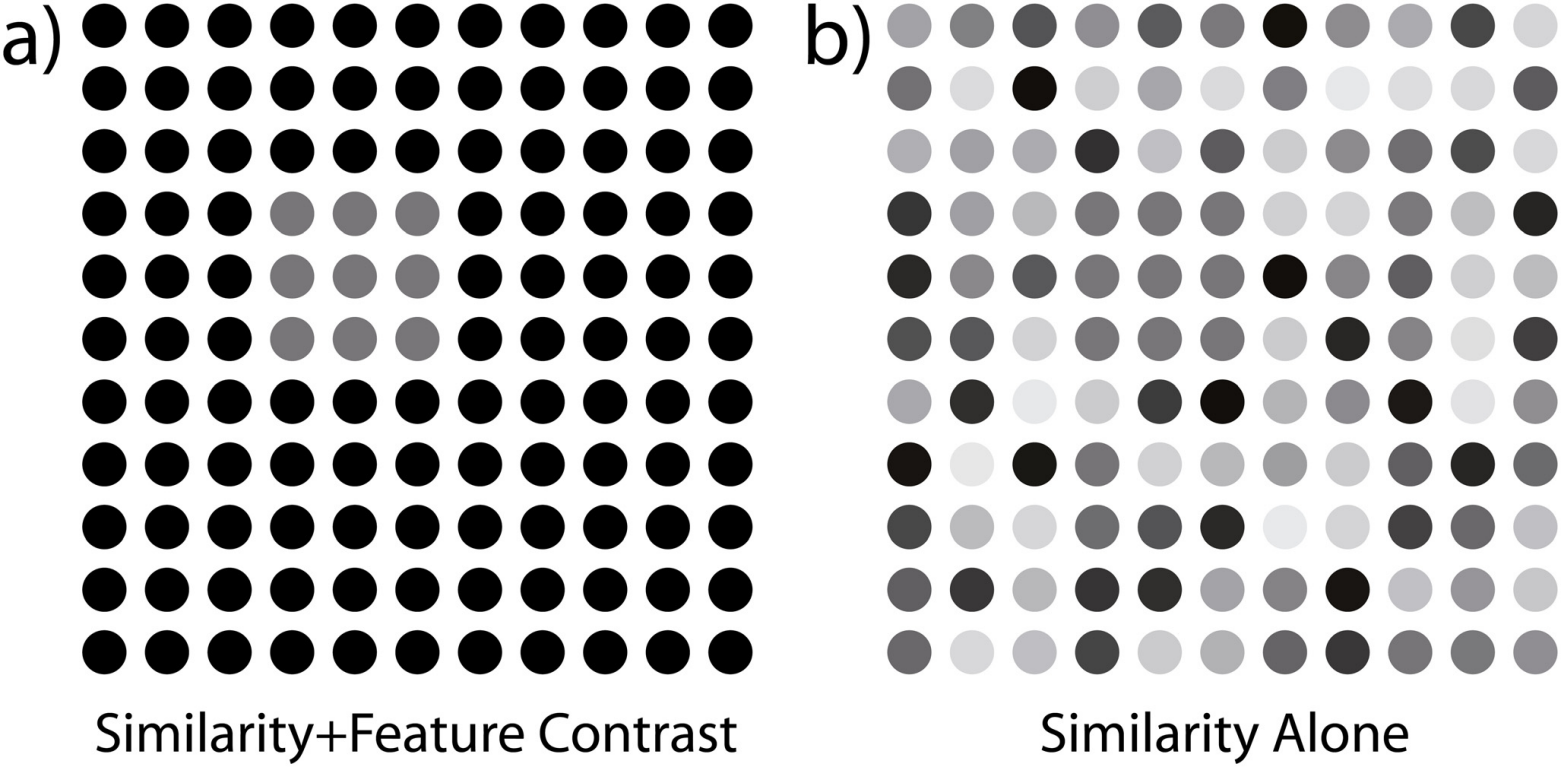
Comparison of grouping cues. (a) illustrates the combination of element similarity and luminance contrast to make the foreground figure visible, (b) removes the feature contrast but adds variability among background elements while keeping the mean luminance of foreground and background equal. Luminance variability is sampled uniformly from the entire interval of available luminance values.

Less attention has been devoted to potentially beneficial effects of grouping principles other than good continuation to contour integration. Among the few studies investigating the combination of grouping cues in contour integration, one found a subthreshold color cue to be scarcely effective in increasing contour integration performance [40]. Similarly scant results were reported for the combination of a motion cue with orientation collinearity [41]. On the other hand, significant performance benefits due to a second cue have been reported for flicker [36] and texture cues [42], when added to an orientation defined contour. Most of the aforementioned studies, however, implemented the additional cue in such a way that it intertwined two sources of perceptual grouping: feature similarity and feature contrast. This resembles the way most textbooks on Gestalt principles illustrate the principle of similarity (see Fig 1a). The foreground figure separates from its background not only due to the homogeneity of the figure elements but also their feature contrast against the background. To eliminate the feature contrast cue and only retain the similarity cue one can add feature variability to the background while keeping the mean feature value of foreground and background equal (Fig 1b). The only contour integration study we are aware of which separated the similarity principle from feature contrast cues showed that contours with homogenous disparities of path elements are visible when embedded in depth heterogenous backgrounds [43]. However, saliency of depth defined contours was significantly lower compared to orientation defined contours.

The present study investigates the contribution of similarity cues to contour integration while dispensing with coincident feature contrast cues. Owing to previous experience [44–46] we chose spatial frequency as a good candidate to establish feature similarity for contours that are already defined by orientation alignment. In line with many association field models [47–50, see, among others,], contour saliency can emerge from three possible sources when the two cues of good continuation and similarity are combined: (1) orientation collinearity among contour elements, (2) similarity grouping due to uniform spatial frequencies of contour elements, and (3) the cooperation between orientation collinearity and similarity grouping. When such cooperation occurs, two principal modes can be distinguished. First, increased saliency may result as a concerted effect of two independent contour detection mechanisms, one operating on collinear orientations (i.e., the association field), the other on element similarity with respect to carrier spatial frequency. Benefits in contour integration performance should in this case be compatible with probability summation among independent neural mechanisms [51]. Alternatively, the saliency gain may be due to intrinsic properties of the contour integration mechanism, such as the reduction of false positives [52], or privileged interconnections between collinear orientation detectors with similar spatial frequency tuning, in which case we expect to find saliency gains larger than predicted by probability summation.

## Materials and Methods

### Sample

16 undergraduate and graduate students (8 female, age range 19–27 years) served as observers, recruited through in-house message boards. Students were paid or received course credits for their participation. Experiments were conducted in 2008/2009. All observers had normal or corrected to normal vision. The students had no former psychophysical experience in contour integration.

### Ethics statement

Prior to the experiment, participants were informed about the course and expected duration of the experiment. They received a general description of the purpose of the experiment but not about specific outcome expectations. All participants signed a written consent form according to the World Medical Association Helsinki Declaration and were informed that they could withdraw from the experiment at any time without penalty. At the time of data collection, no local ethics committee was instated. Noninvasive experimental studies without deception did not require a formal ethics review provided the experiment complied with the relevant institutional and national regulations and legislation which was carefully ascertained by the authors. After completing the experiment, a summary of their individual data was shown to the observers and the results pattern explained within the scope of the purpose of the study.

### Stimuli

Stimulus displays consisted of approximately *n*_*C*_ + *n*_*BG*_ = 170 Gabor micropatterns defined by

$$\begin{array}{c} g(x,y)=\exp(-\frac{x^{2}+y^{2}}{2\sigma^{2}})\sin(2\pi f(x\cos(\omega )-y\sin(\omega ))). \end{array}$$ (1)

In (Eq 1) let $\omega =\frac{\varphi }{180}\pi$, with *φ* the rotation angle measured in degrees. Spatial phase altered randomly between even and odd sines. Gabor micropatterns were spatially limited to a diameter of 1.4° visual angle by setting the standard deviation *σ* of the Gaussian envelope to 0.28° and clipping beyond a radius of 2.5 *σ*-units. Random sampling of carrier spatial frequencies *f* started at values not lower than 1.0 cycles per degree (cpd) in all experimental conditions. The defined Gabor size thus kept bandwidths of low frequency micropatterns confined within reasonable intervals.

**Fig 2:**
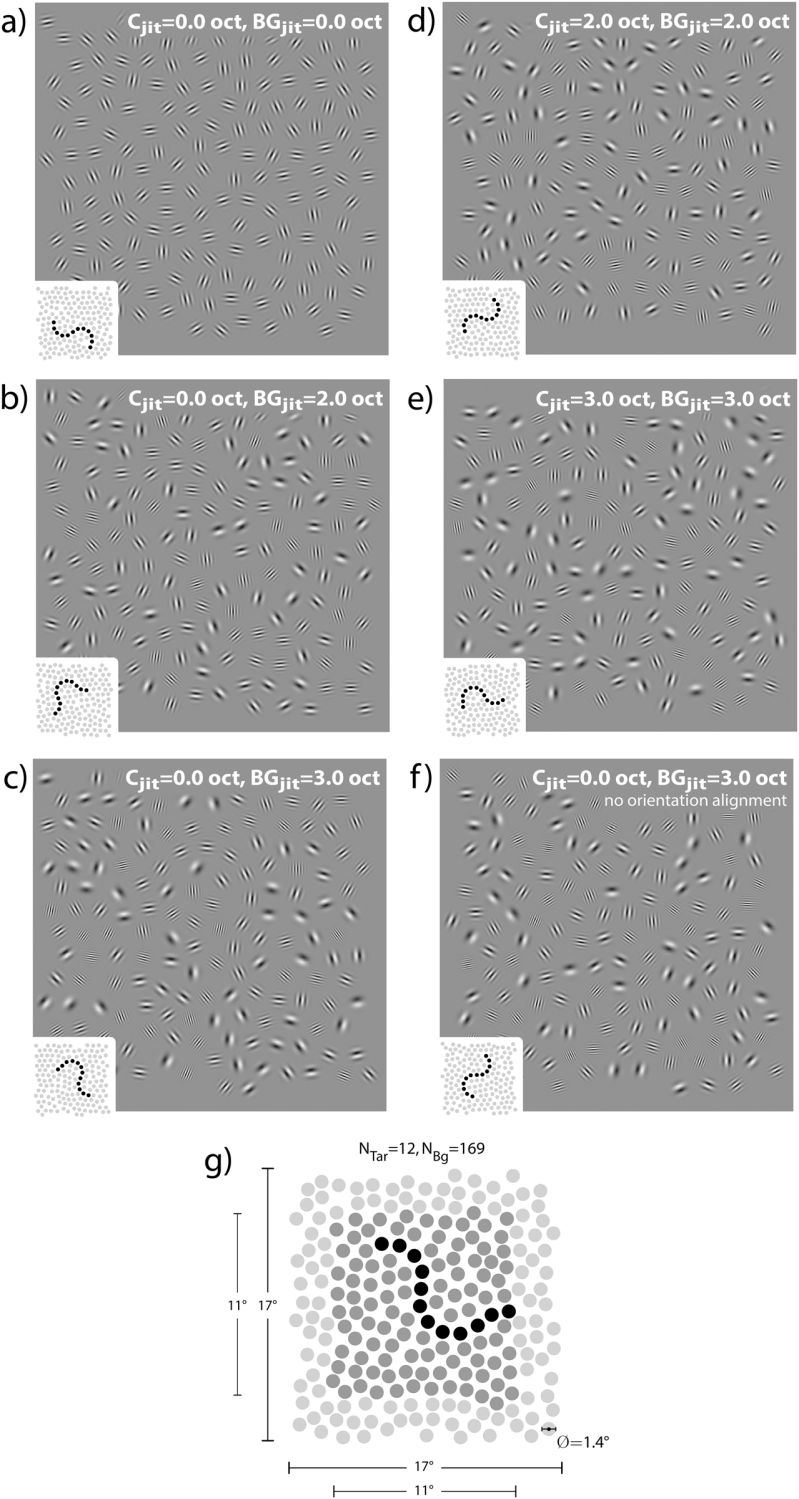
Stimuli used in the experiment. Iconograms depict the location of the target contour (black dots) in the background field (grey dots). Elements were either completely homogenous or jittered by 2.0 octaves, or 3.0 octaves, respectively. Five combinations of contour “C” and background “BG” carrier spatial frequency jitter were realized, as depicted in panels (a) through (e). All contours are assigned maximum possible visibility by setting the tilt angle to *θ* = 0. The last panel (f) illustrates one of the control conditions. All elements in both the background and along the contour have random orientations, contour elements are homogenous in carrier spatial frequency while background elements exhibit jitter (3.0 octaves in the example shown here). Contour saliency may thus emerge only due to spatial frequency homogeneity. The expected value of the spatial frequency distribution in all jitter conditions is $\bar{f}=3.36$.

Target contours encompassed *n*_*C*_ = 12 Gabor elements with an inter-element angle of ±30°, embedded in fields of randomly oriented background elements. Contour shapes ranged from a regular or mirrored “S” to a “U” type figure with outwardly bound tails. Fig 2 shows examples of possible contour shapes. The bounding box of a contour always fell within a 11° × 11° square region around the center of the whole 17° × 17° background lattice (see Fig 2g). This limitation was introduced to prevent contours from extending too far into the peripheral field where contour integration is known to cease [53].

Construction of target stimuli fell into four steps. First, a contour path was constructed as a smooth trajectory of disjoint elements [6]. Second, background positions were arranged as a hexagonal grid and superimposed onto the contour. Background elements that overlapped contour elements were removed from the grid. Third, a perturbation method [54] was used to randomly displace background element positions. Finally, Gabor elements were placed on all positions. Each background element was assigned a random orientation while contour elements were initially coaligned with the global path trajectory. Carrier spatial frequencies were sampled for both background and contour elements depending on experimental condition. Creation of distracter stimuli adhered to the same procedure, only with the elements of the embedded contour also assuming random orientations.

Contour integration research relies on the assumption that stimuli are artifact-free with respect to spatial characteristics of contour and background element positions [55, 56]. Pairwise euclidian distances between adjacent contour elements therefore jittered according to the probability distribution of inter-element distances in the background. This distribution was estimated prior to the experiment from Delaunay triangulations [57] of 25.000 patterns containing only background positions. Post-hoc analysis of stimuli used during the experiment showed that stimuli exhibited near identical distributions of contour-to-contour, contour-to-background, and background-to-background element distances. Hence, not only were first-order density cues practically absent from our stimuli [58] but the whole distribution of spatial distances of contour element positions approximated that of background elements.

Orientation jitter in the background was always maximal, sampling *φ* uniformly from the interval [0°;360°]. Detectability of target contours was then modulated by rotating the orientation of Gabor elements away from perfect collinearity by a given tilt angle *θ*. Increasing values of *θ* produce perceptually more and more jagged contours and diminish contour detection performance while keeping the global curvature of the path intact. The absolute value of *θ* was fixed for any particular contour visibility level, with only the sign alternating randomly. In accordance with observations from previous studies, contours such as used in our experiment become undetectable at about ∣*θ*∣ ≥ 30° [59].

### Spatial Frequency Variation

There were five conditions of spatial frequency jitter plus two supplemental conditions controlling for saliency effects due to spatial frequency similarity alone. To have spatial frequency jitter follow a perceptually uniform random distribution we defined spatial frequencies on an octave scale as *f* = 2^*u*^. Depending on experimental condition, the parameter *u* was sampled from a uniform distribution with varying lower and upper limits. Limits were chosen such that the expected value of the resulting exponential distribution was $\bar{f}=3.36$ cpd in all cases. Stimulus examples for the five spatial frequency jitter conditions with orientation defined contours are shown in Fig 2a–2e, one of the control conditions with similarity defined contours is depicted in Fig 2f.

- **C**_0.0_ **BG**_0.0_—Spatial frequency was constant for both contour and background elements (*f*_*C*_ = *f*_*BG*_ = 3.36 cpd).
- **C**_0.0_ **BG**_2.0_—Spatial frequency was constant along the contour (*f*_*C*_ = 3.36) and randomly sampled for background elements spanning a 2.0 octaves wide interval ($f_{BG}=[1.\bar{5};6.\bar{2}]$ cpd).
- **C**_0.0_ **BG**_3.0_—Same as the previous condition but with background jitter spanning a 3.0 octaves wide interval (*f*_*BG*_ = [1;8] cpd).
- **C**_2.0_ **BG**_2.0_—Spatial frequencies were randomly sampled from a 2.0 octaves wide interval for both contour and background ($f_{C}=f_{BG}=[1.\bar{5};6.\bar{2}]$ cpd).
- **C**_3.0_ **BG**_3.0_—Same as the previous condition but with a 3.0 octaves wide jitter range (*f*_*C*_ = *f*_*BG*_ = [1;8] cpd).
- **Controls**—${\text{C}}_{0.0}^{*}{\text{BG}}_{2.0}$ and ${\text{C}}_{0.0}^{*}{\text{BG}}_{3.0}$ were the two control conditions, again with either a 2.0 or 3.0 octaves wide sampling interval for carrier spatial frequencies of background elements. Contour elements had homogenous spatial frequencies but orientations randomly tilted away from perfect collinearity. Hence, in both control conditions, contour detection could no longer rest on orientation alignment but only on spatial frequency similarity among contour elements.

### Elimination of artificial target cues

In conditions with spatial frequency jitter along the contour, at least every third contour element was algorithmically set to deviate from its predecessor by more than $\frac{3}{4}$ of the maximum spatial frequency jitter range. This prevented an accidental formation of long chains of elements with similar spatial frequency values along the contour.

During pretesting we noticed that in both control conditions many subjects managed to detect a target stimulus significantly above chance level while being incapable of reporting the actual shape of the contour. Further testing revealed that subjects based responses on the perception of unusually high densities of mid-range spatial frequencies (i.e., around 3.36 cpd) in the potential target area which could only occur in target stimuli. To counter this artificial target saliency, we devised a twofold solution. First, for each distracter stimulus appearing in C_0.0_BG_2.0_ and C_0.0_BG_3.0_ trials, *n* = 12 random Gabor elements within the target area were assigned a spatial frequency of *f* = 3.36 cpd. Second, prior to each stimulus presentation, a spatial frequency shift value was randomly sampled from an interval of [0.0;0.25] octaves and added to all Gabor elements in the respective stimulus, thereby increasing observer uncertainty about the overall spatial frequency mean across stimulus presentations.

### Apparatus

Stimuli were generated on a ViSaGe system manufactured by Cambridge Research Systems and displayed on a Samsung 959NF color monitor. The mean luminance of the screen was 50 cd/m^2^. Stimuli were displayed with a fixed Michelson contrast of 0.95. Color values were taken from a linear grey staircase consisting of 255 steps chosen from a palette of 4096 possible grey values. The relation between grey level entries and the luminance on the screen was linearized by means of gamma correction tables. Linearity was checked before the experiment using a Cambridge Research Systems ColorCAL colorimeter. The determination coefficient of the regression line exceeded *r* ^2^ = .98 in all cases. The refresh rate of the monitor was 80 Hz at a horizontal frequency of 84.62 kHz, the pixel resolution was set to 1348 × 1006 pixels. The ambient illumination of the darkened room approximately matched the illumination on the screen. Patterns were viewed binocularly at a distance of 70 cm. Subjects used a chin rest for head stabilization and gave their responses with their dominant hand via an external response keyboard.

### Psychophysical task

A 2AFC contour detection task was used. Subjects saw two subsequent stimulus frames. With a button press subjects indicated whether the first or the second frame contained a contour. Acoustical feedback about correctness was provided by a brief tone signal. Stimulus frame presentation was terminated by masking with spatial noise at a grain resolution of 3 pixels. The temporal order of events was fixation (750 msec)—SOA (400 msec)—1st stimulus frame (300 msec)—mask (400 msec)—SOA (400 msec)—2nd stimulus frame (300 msec)—mask (400 msec)—blank frame until response.

### Preliminary measurements and main experiment

Measurement of contour integration performance proceeded in two steps, both of which employed the method of constant stimuli. Initially, contour detection was measured individually for each experimental condition and subject. Detection rates were recorded on five tilt angles *θ* at 32 repetitions, and fitted with a Gaussian distribution function of the general form

$$\begin{array}{c} F(\theta ;\mu_{\theta },\sigma_{\theta })=1-\frac{1}{4}[1+\text{erf}(\frac{\theta -\mu_{\theta }}{\sqrt{2}\sigma_{\theta }})] \end{array}$$ (2)

where erf denotes the error function. The two parameters *μ*_*θ*_ and *σ*_*θ*_ were estimated with the Levenberg-Marquardt algorithm [60]. The parameter *μ*_*θ*_ of the Gaussian distribution function defined in (Eq 2) serves as a direct estimate of the.75 detection threshold from 2AFC measurements, the parameter *σ*_*θ*_ is inversely proportional to the slope of the psychometric function, with smaller values of *σ*_*θ*_ indicating steeper psychometric curves. Note that estimated detection performance *F*(*θ*) is supposed to decrease with increasing tilt angle *θ*. Hence, a *higher* threshold value *μ*_*θ*_ denotes *better* visibility of a contour since it retains saliency until higher tilt values.

Two psychometric functions with sufficient fit were obtained from each condition and subject, whereupon individual sets of five tilt angles were extrapolated. Tilt angles were selected to yield detection rates of 0.59, 0.67, 0.75, 0.83, and 0.91 for each subject in the respective condition. In the main experiment these sets of tilt angles for all experimental conditions were randomly intermixed at 32 repetitions for each angle. The implementation of a main experiment that, in principle, differed from the preliminary measurements only by complete randomization of trials may seem superfluous at first glance. We nonetheless opted for a dedicated main experiment in order to preempt any response bias, stemming from condition specific detection strategies devised by subjects. Analyses, however, proved that the pattern of results from the main experiment closely reflected results from calibration measurements, only at a slightly lower overall performance level. Data analyses rest on two performance measures, tilt angle threshold *μ*_*θ*_, defined as the 0.75 point of the psychometric function, and the standard deviation *σ*_*θ*_ of said function.

Measuring detection rates at 5 tilt angles, each with 32 replications for 5 target conditions plus two control conditions with only one random tilt angle had each subject undergo 864 trials in the main experiment. Together with the preliminary measurements each subject completed at least 2464 trials during the whole experiment. Trials were administered in two sessions on separate days with sufficient warmup trials and one brief intermittent pause. Annotated analysis data are provided in file S1 Dataset.

## Results

### Psychometric functions

**Fig 3:**
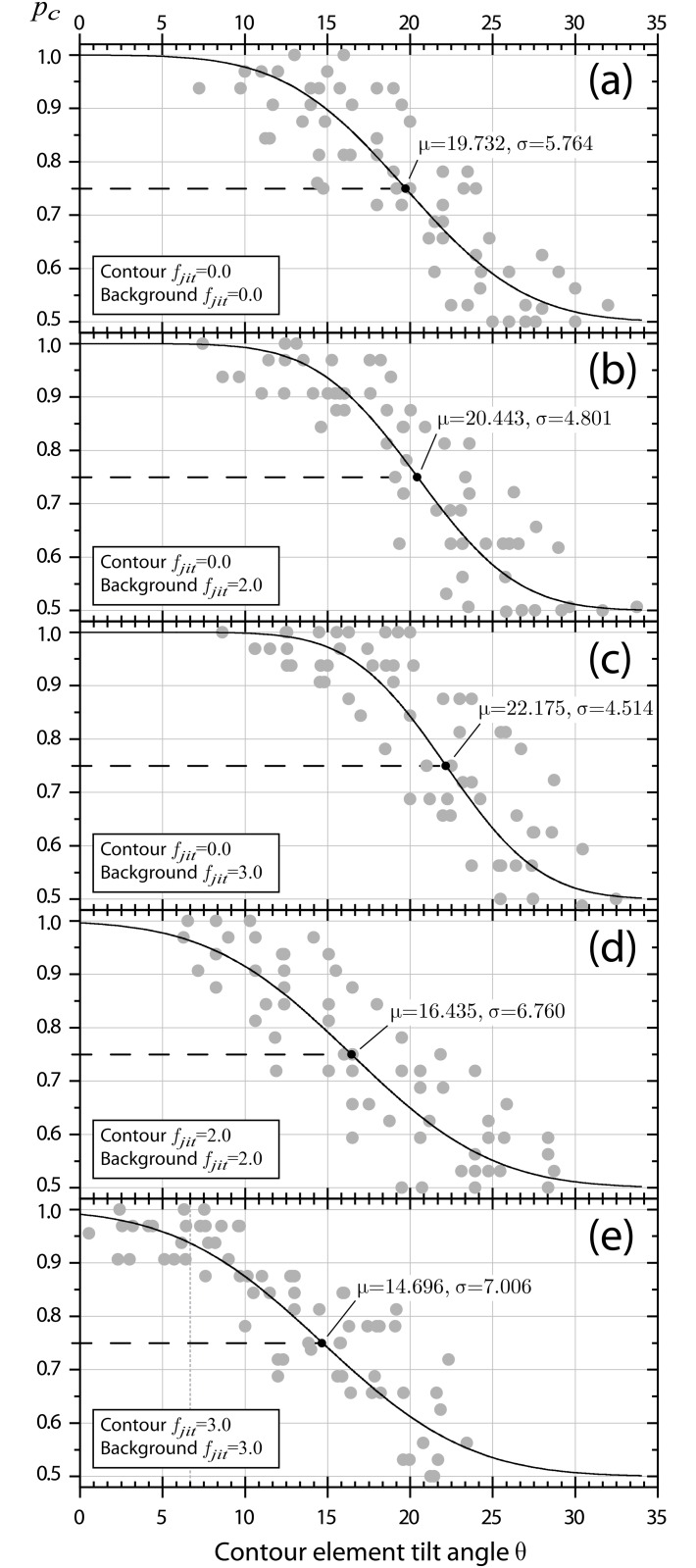
Psychometric functions. Fits are depicted separately for all five combinations of contour and background spatial frequency jitter. Data points represent proportion correct measures from all subjects at multiple tilt angles *θ*. For each experimental condition a summary psychometric curve is drawn (solid line). The curve is a cumulative Gaussian function intersecting at the between subject mean tilt angle threshold, $\bar{\mu_{\theta }}$, and with a mean standard deviation estimate, $\bar{\sigma_{\theta }}$. Mean estimates are highlighted by black dots.

Psychometric function data from the main experiment are summarized in Fig 3. Data points represent proportion correct values for all subjects at the respective tilt angle *θ*. A generalized psychometric function is displayed with parameters *μ*_*θ*_ and *σ*_*θ*_ averaged across subjects.

Mean estimates of *μ*_*θ*_ and *σ*_*θ*_ follow a consistent pattern. Saliency of frequency homogenous contours amplifies with increasing spatial frequency jitter in the surround, while at the same time psychometric functions become steeper, as can be seen in Fig 3a and 3c). When spatial frequency jitter is introduced for contour elements as well, both the contour saliency and the psychometric function slope gradually reduce again, as illustrated by Fig 3a, 3d and 3e).

### Statistical testing

**Fig 4:**
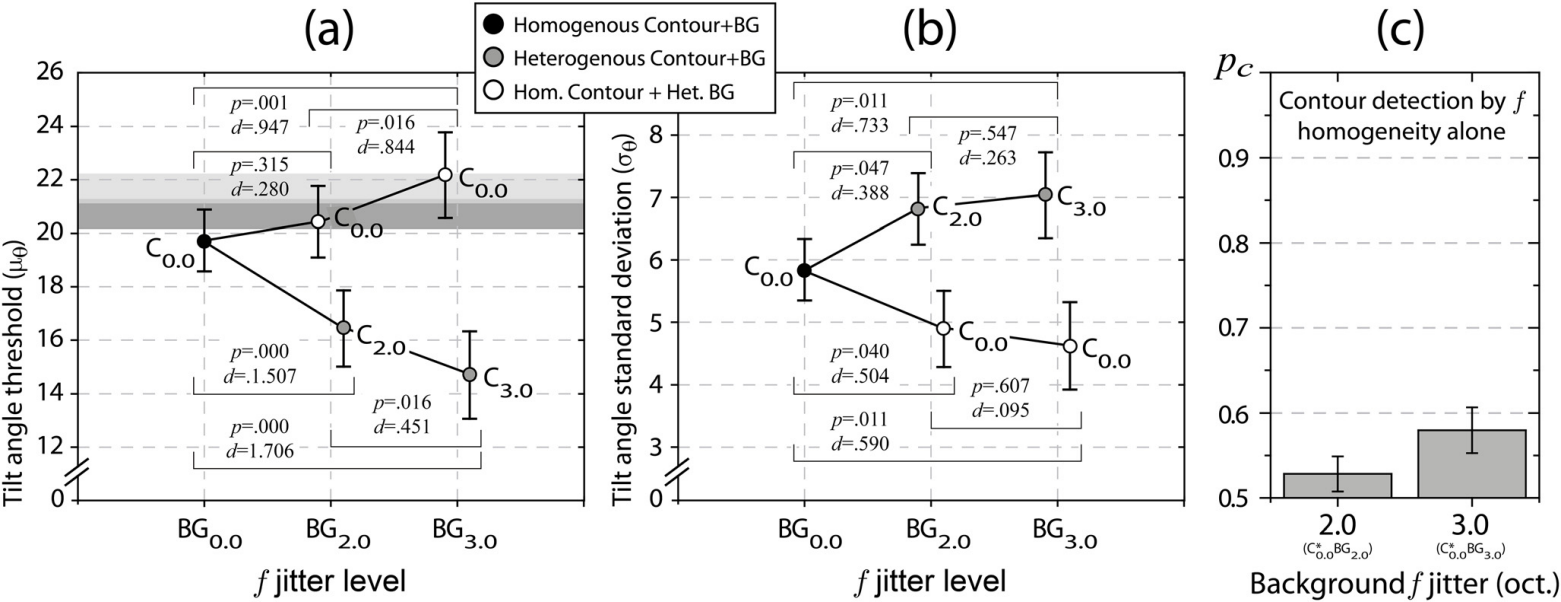
Main effects from rmANOVA. Depicted are between subject means (a) for tilt angle threshold estimates *μ*_*θ*_, and (b) standard deviation estimates *σ*_*θ*_ for all five combinations of contour (C) and background (BG) carrier spatial frequency jitter. Subscripts denote the jitter level in octaves. The dark grey area represents the upper half of the 95% confidence interval for the probability summation prediction for the 2.0 octaves jitter condition, the light gray area for 3.0 octaves, respectively (see text). Panel (c) shows mean proportion correct rates for the detection of contours defined by just spatial frequency similarity, embedded in backgrounds with spatial frequency jitter of 2.0 or 3.0 octaves magnitude, together with their 95% confidence intervals.

Tilt angle threshold and standard deviation estimates derived from psychometric function data were analyzed by separate repeated measures ANOVAs (rmANOVA). The five variants of contour and background spatial frequency jitter served as repeated measures factor levels. The two control conditions is analyzed at a later stage. Table 1 summarizes the results of the univariate analyses for both dependent measures, Fig 4 illustrates the major findings.

**Table 1:** rmANOVA results for tilt angle thresholds *μ*_*θ*_ and tilt angle standard deviations *σ*_*θ*_.

	a) Tilt angle threshold *μ*_*θ*_				b) Tilt angle sd *σ*_*θ*_			
Source	${\hat{\sigma }}^{2}$	*df*	*F*	*p*	${\hat{\sigma }}^{2}$	*df*	*F*	*p*
*f* Jitter	149.340	4	37.916	.000	20.098	4	11.137	.000
Error_within_	3.940	60			1.805	60		
Error_between_	20.820	15			3.296	15		

Summarized are the source of variation, estimated population variances, degrees of freedom, the *F*-statistic, and the significance level. Regardless of insignificant Mauchly tests (*χ* ^2^(9) = 13.088, *p* = .159 for *μ*_*θ*_; *χ* ^2^(9) = 8.287, *p* = .505 for *σ*_*θ*_), degrees of freedom were Huynh-Feldt corrected before calculating p-values (see Lecoutre, 1991).

#### Detection performance

Jitter conditions exert a highly significant effect on contour integration performance (Table 1a). Contour visibility increases when spatial frequency jitter is added to background elements but not contour elements (see Fig 4a). While background jitter of 2.0 octaves does not suffice to elevate thresholds by a statistically significant margin, visibility of homogenous contours significantly benefits from 3.0 octaves spatial frequency jitter in the background. Planned pairwise contrasts of C_0.0_BG_3.0_ are significant against both previous jitter levels (C_0.0_BG_2.0_ and C_0.0_BG_0.0_), each with high effect sizes. On the other end, contour detection thresholds suffer significantly from spatial frequency jitter along the contour. All planned pairwise contrasts between C_0.0_BG_0.0_, C_2.0_BG_2.0_, and C_3.0_BG_3.0_ reach significance with moderate to very high effect sizes (Fig 4a). Modulation of tilt angle standard deviations depending on jitter condition is highly significant (Table 1b). Increasing the spatial frequency jitter along the contour attenuates the slope of psychometric functions (Fig 4b). Planned pairwise comparisons prove both differences between the conditions with spatial frequency jitter (C_2.0_BG_2.0_ and C_3.0_BG_3.0_) against the homogenous condition (C_0.0_BG_0.0_) statistically significant with moderate to high effect sizes. In contrast, leaving the spatial frequency of contour elements constant and introducing jitter only in the surround leads to steeper psychometric curves. Both conditions in question (C_0.0_BG_2.0_ and C_0.0_BG_3.0_) yield significantly higher slopes when compared to the fully homogenous condition. Effect sizes are moderate (see Fig 4b).

#### Saliency gains

This raises the question whether the observed saliency advantages of homogenous contours in heterogenous surrounds root in specific architectural properties of neural contour integration networks, or may be explained simply by probability summation due to simultaneous integration of orientation aligned contours and grouping of similarity defined contours. The control conditions are a viable means to decide between the two alternatives, for they provide an estimate about contour visibility based on spatial frequency similarity alone. Results from the two control conditions are depicted in Fig 4c. Detection rates of similarity defined contours are very low, yet not entirely at chance level. Hence, spatial frequency similarity might contribute to the visibility of orientation defined target contours as an independent source of saliency. In such a case of probability summation, a prediction of detection performance can be derived. Let *F*_0_(*θ*) be the detection rate of a homogenous contour with a given tilt angle *θ* in a homogenous background, and *G*(*u*) the detection rate of a homogenous (i.e., similarity defined) contour embedded in a background with spatial frequency jitter of *u* octaves. I The detection rate predicted by probability summation after guessing correction then computes as

$$\begin{array}{c} {\tilde{F}}_{0}(\theta ;u)=1-[(1-F_{0}(\theta ))(1-G(u))]. \end{array}$$ (3)

Both *F*_0_(*θ*) and *G*(*u*) are available from our data. *F*_0_(*θ*) is the psychometric function estimate from condition C_0.0_BG_0.0_, and *G*(*u*) is the detection rate from the respective control condition (*u* = 2.0 or *u* = 3.0 octaves), both corrected for guessing probability. By setting (Eq 3) equal to.5 and solving for *θ*, one arrives at a probability summation estimate for the tilt angle threshold in the C_0.0_BG_2.0_ and C_0.0_BG_3.0_ conditions. From there, confidence interval limits around the threshold predictions can be constructed based on the standard error of the original estimate of *μ*_*θ*_.

These confidence intervals are shown as gray shaded areas in Fig 4a. It is apparent that the threshold elevation for both conditions of homogenous contours in heterogenous surrounds (C_0.0_BG_2.0_ and C_0.0_BG_3.0_), when compared to wholly homogenous contour stimuli (C_0.0_BG_0.0_), is compatible with the assumption of probability summation between an alignment based contour integration mechanism and an independent spatial frequency similarity detector.

#### Joint analysis

**Fig 5:**
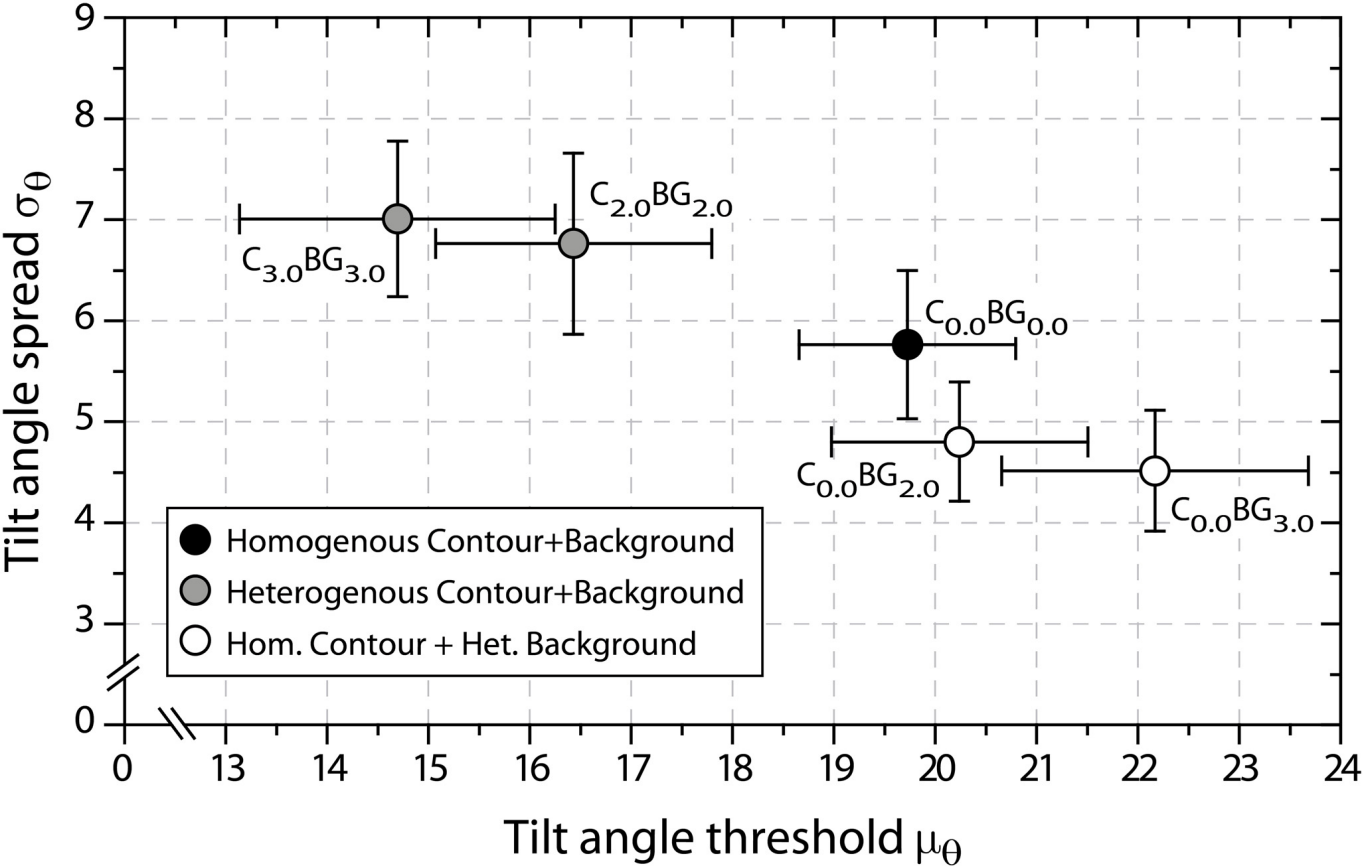
Joint effects of spatial frequency jitter on tilt angle threshold and tilt angle standard deviation. Panel (a) depicts centroids defined by tilt angle threshold *μ*_*θ*_ (*x*-component) and standard deviation estimate *σ*_*θ*_ (*y*-component), together with the 95% confidence limits of each component. p-Values are taken from planned multivariate contrasts between the connected centroids.

The course of main effects in Fig 4a and 4b for the tilt angle threshold and standard deviation estimates suggests a systematic relationship between the two measures. A multivariate analysis of variance (MANOVA) was conducted to assess the effect of spatial frequency jitter on the two dependent measures. MANOVA results were highly significant (Wilk’s *λ* = 0.026;*F*(8,8) = 36.836, *p* = 0.000), supporting the clear pattern that emerges from the biplot in Fig 5. With increased spatial frequency jitter among background elements distances to the homogenous condition (C_0.0_BG_0.0_) become larger. For conditions with homogenous contours embedded in heterogenous surrounds, this is due to steeper psychometric functions and higher tilt angle thresholds, whereas for conditions with fully heterogenous spatial frequencies psychometric functions slopes decline and detection thresholds attenuate. All planned pairwise multivariate contrasts reach statistical significance except for the comparison between the two fully heterogenous stimulus conditions (C_2.0_BG_2.0_ against C_3.0_BG_3.0_).

To estimate the efficiency of a detector system, the “variation coefficient” $\rho =\frac{\sigma }{\mu }$ can be computed as the ratio of standard deviation to mean parameter [61]. If the variation coefficient is constant across different mean parameters, the standard deviation of the underlying distribution rises proportional to the mean parameter. This proportionality is a common behaviour of many psychometric variables, such as mental test scores [62]. In sensory analysis constancy of the variation coefficient was observed in simple discrimination tasks under various conditions [63, 64], indicating a detector that operates at a constant signal to noise ratio [65]. Larger values of the variation coefficient imply stronger internal noise at the detection criterion, and therefore less efficient detection [66]. An important implication of constancy of the variation coefficient is parallelism of psychometric functions on log-intensity scales [67]. If the Weibull model is used for the psychometric curve, log-parallelism is obtained if the slope parameter *β* is constant. In addition, regardless of whether the Weibull model or a Gaussion model is used for the psychometric curve, constancy of the variation coefficient implies log-parallelism, indicating the same detector efficiency over a range of stimulus parameters [66]. Analysing the slope parameter for various cases that go beyond a linear systems approach can distinguish between plausible cases of sensory integration and decisional strategy [68], i.e., channel uncertainty and probability summation among channels with additive and multiplicative noise. Since the slope parameter can also be used to estimate the power of the nonlinear signal transducer [69, 70], it is widely used to evaluate detection models that involve signal uncertainty [71].

**Fig 6:**
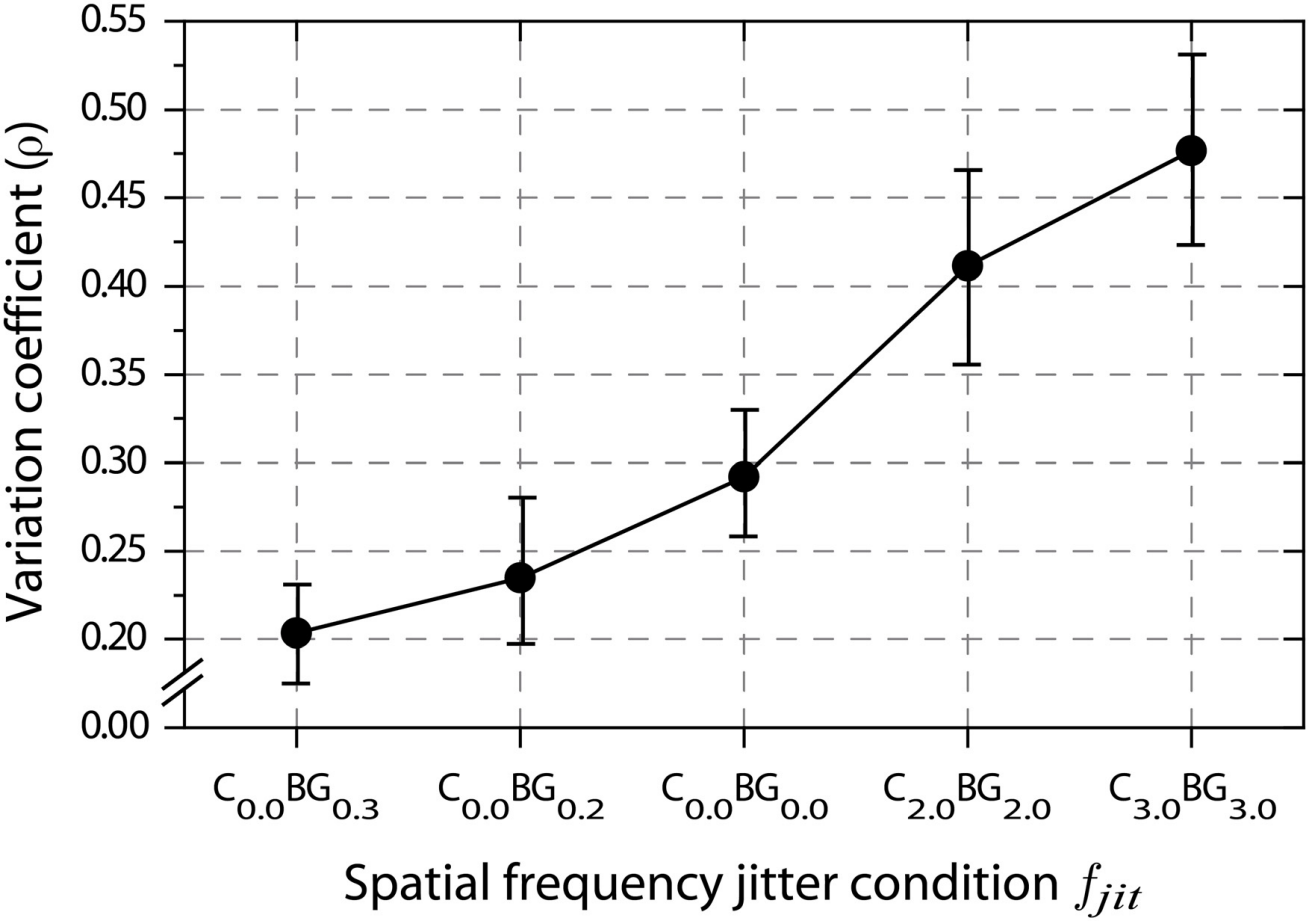
Variation coefficients. Coefficients are computed as $\rho =\frac{\sigma_{\theta }}{\mu_{\theta }}$. The parameters *σ*_*θ*_ and *μ*_*θ*_ are taken from psychometric function fitting, as described in methods.

Applied to our data, the variation coefficient can capture an important relationship between psychometric function slope and threshold. If an additional similarity cue yields a higher certainty in detecting orientation defined contours, the increased signal-to-noise ratio should result in higher slopes of psychometric curves for the same mean parameters and thus smaller variation coefficients. Spatial frequency jitter along the contour on the other hand ought to impair detection certainty and result in shallower psychometric functions. The uncertainty arising from the necessity of monitoring multiple spatial frequency bands across a large stimulus field for potential target contours has long been known to reduce performance remarkably [72, 73]. Fig 6 proves the importance of feature uncertainty in contour detection experiments. A sharp increase of the variation coefficient *ρ* occurs for conditions with spatial frequency noise along the contour, indicating a disadvantageous signal (i.e., contour) to noise (i.e., background) relation in such stimuli. In contrast, when similarity is available as a second cue to an alignment defined contour, variation coefficients become smaller, denoting a gain in certainty about contour presence or absence. This gives rise to the conclusion that the similarity principle not so much affects the contour detection process itself but primarily reduces observer uncertainty about whether a potential candidate really is a contour or just a false positive.

## Discussion

Results show that contour integration based on orientation collinearity does benefit from similarity cues but only in orders of magnitude expected from probability summation between independent saliency mechanisms. This suggests functional independence of the two involved grouping mechanisms. One mechanism integrates contour elements based on orientation collinearity, the other evaluates similarity relations among stimulus patches. Such functional independence of the contour integration mechanism from other saliency generating processes has already been found in previous experiments on the combination of orientation alignment with supplemental cues such as motion [41] and color [74]. It shall be noted, though, that the mean tilt angle threshold for the high jitter condition lies in close proximity to the upper bound of the probability summation prediction.

Variability of spatial frequencies along the contour not only affects detection thresholds but the steepness of psychometric functions. The certainty at which contours are detected deteriorates with increasing jitter along the contour, and grows when similarity cues are added to the contour. Spatial frequency similarity among contour elements in cluttered background apparently reinforces the observer’s decision about contour presence in a stimulus. Considering our stimulus arrangements, it can be reasoned that a potential benefit for the integration of homogenous contour elements in surrounds with spatial frequency variation is overshadowed by higher feature uncertainty of the observer, who needs to scrutinize the stimulus for potential contour candidates in multiple spatial frequency bands simultaneously. The penalty in target detection due to the spread of attention across multiple spatial frequency bands is known to reduce performance significantly [72, 75], and might thus have mediated certainty conditions at the decision stage. Our data support this argument. Although spatial frequency similarity fails to facilitate contour integration by a margin beyond probability summation, it does exert a striking effect on psychometric function slopes. When both contour defining principles—similarity and orientation collinearity—are combined along the contour, psychometric functions steepen, and flatten when contour elements jitter in carrier frequencies, as reflected by the variation coefficient.

Despite its potentially beneficial effects on observer certainty, similarity as such does not qualify as a particularly strong figure cue on its own. In accordance with earlier findings [39] contours defined by spatial frequency similarity in heterogenous surrounds proved nearly invisible, with detection rates only barely exceeding chance level even in the highest jitter conditions. We take that as evidence to suggest the absence of specialized cortical networks for similarity driven form completion in cluttered images, rendering local orientation collinearity the key feature for establishing contour saliency.

Finally, our results corroborate earlier findings showing that contour integration is possible across large spatial frequency disparities between adjacent contour elements [76]. Detection thresholds in our study attenuate by about 3° for spatial frequency jitter of two octaves and 5° at three octaves but contours remain well visible in general. Even under the highest jitter conditions, tilt angle thresholds remain at levels above 10°. Taken together with the global contour curvature of ±30°, this means that contour integration is operational even with spatial frequency disparities up to 3.0 octaves and large orientation differences between adjacent contour elements. This is in line with the assumption that orientation information is integrated efficiently with dynamic orientation bandwidth [77] across a wide range of spatial frequency [78]. Psychometric function data further reveal that for small tilt angles, contour integration approaches perfect performance for almost all subjects even in the highest spatial frequency jitter conditions. These figures reinforce the notion of contour integration as a robust shape detection mechanism [39] which integrates not only over a broad range of spatial frequencies but, as recently shown, also over other disruptive factors like large inter-element distances, temporal flicker, or colour and luminance disparities [79].

To link adjacent segments of coaligned stimulus elements, the contour integration mechanism may exploit the outputs of orientation detectors with broad spatial frequency tuning, or, alternatively, sum over the outputs of multiple scale-selective detector populations [27]. There is ample evidence that area V1 harbors all necessary means for full-fledged contour integration [12, 79, 80], possibly aided by feedback connections from higher visual areas [81, 82]. Moreover, saliency benefits due to multiple cues can be explained by low-level mechanisms for bottom-up salience located as early as V1 [83]. A variety of studies have, however, extended our understanding of contour integration by highlighting specific characteristics of human contour integration which might exceed the functional properties of V1 cells. Contour integration is binocular in nature [84], remains functional when presented under conditions of binocular disparity between contour elements [38, 43], combines multiple scales [39, 76], and works on stimuli devoid of luminance modulation such as collinear bars of isoluminant color [85]. Integration across spatial frequency, across eyes, and through color hints at the contribution of other visual areas besides V1. One candidate is area V2 where cells with all necessary properties have been found [86]. Electrophysiological and neuroimaging studies have further diversified our picture of the locus of contour integration. Their findings implicate ventral regions like the parietal cortex, inferio-temporal cortex (IT), and the lateral occipital complex (LOC) in the grouping of contour parts into outlines of figures and shapes on larger scales [30, 32, 87]. Recent studies attempted to reconcile the different, partly opposing views of early versus late implementation of contour integration routines. Contour dependent activation appears to be tightly coupled between multiple cortical levels. While an initial peak of contour related activation has been found in area V4, delayed but congruent activation followed on V1, probably modulated by inter-area feedback projections [31]. Taken together, it is likely that contour integration and form completion involve multiple processing levels with mutual interactions along the neural processing stream [88–90]. The results of the present study help to highlight specific characteristics of such a contour integration mechanism with respect to the effects of spatial frequency variation.

## Supporting Information

S1 Dataset The files included in the ZIP archive provide the dataset on which all statistical analyses were conducted, annotations to connect the dataset with the names and labels used in the article, and a legend to explain naming conventions. (ZIP)
